# Plasma Exosomes Transfer miR-885-3p Targeting the AKT/NFκB Signaling Pathway to Improve the Sensitivity of Intravenous Glucocorticoid Therapy Against Graves Ophthalmopathy

**DOI:** 10.3389/fimmu.2022.819680

**Published:** 2022-02-21

**Authors:** Jingxue Sun, Jiaxing Wei, Yaguang Zhang, Jingjing Li, Jian Li, Jiazhuo Yan, Min Guo, Jun Han, Hong Qiao

**Affiliations:** ^1^ Department of Endocrinology and Metabolism, The Second Affiliated Hospital of Harbin Medical University, Harbin, China; ^2^ Department of Gynecological Radiotherapy, Harbin Medical University Cancer Hospital, Harbin, China; ^3^ Department of Endocrinology and Metabolism, The Fourth Affiliated Hospital of Harbin Medical University, Harbin, China

**Keywords:** Graves ophthalmopathy, glucocorticoid sensitivity, plasma exosomes, miR-885-3p, AKT/NFκB signaling pathway

## Abstract

Graves ophthalmopathy (GO), a manifestation of Graves’ disease, is an organ-specific autoimmune disease. Intravenous glucocorticoid therapy (ivGCs) is the first-line treatment for moderate-to-severe and active GO. However, ivGCs is only effective in 70%–80% of GO patients. Insensitive patients who choose 12-week ivGCs not only were delayed in treatment but also took the risk of adverse reactions of glucocorticoids. At present, there is still a lack of effective indicators to predict the therapeutic effect of ivGCs. Therefore, the purpose of this study is to find biomarkers that can determine the sensitivity of ivGCs before the formulation of treatment, and to clarify the mechanism of its regulation of ivGCs sensitivity. This study first characterized the miRNA profiles of plasma exosomes by miRNA sequencing to identify miRNAs differentially expressed between GO patients with significant improvement (SI) and non-significant improvement (NSI) after ivGCs treatment. Subsequently, we analyzed the function of the predicted target genes of differential miRNAs. According to the function of the target genes, we screened 10 differentially expressed miRNAs. An expanded cohort verification showed that compared with NSI patients, mir-885-3p was upregulated and mir-4474-3p and mir-615-3p were downregulated in the exosomes of SI patients. Based on statistical difference and miRNA function, mir-885-3p was selected for follow-up study. The *in vitro* functional analysis of exosomes mir-885-3p showed that exosomes from SI patients (SI-exo) could transfer mir-885-3p to orbital fibroblasts (OFs), upregulate the GRE luciferase reporter gene plasmid activity and the level of glucocorticoid receptor (GR), downregulate the level of inflammatory factors, and improve the glucocorticoid sensitivity of OFs. Moreover, these effects can be inhibited by the corresponding miR inhibitor. In addition, we found that high levels of mir-885-3p could inhibit the AKT/NFκB signaling pathway, upregulate the GRE plasmid activity and GR level, and downregulate the level of inflammatory factors of OFs. Moreover, the improvement of glucocorticoid sensitivity by mir-885-3p transmitted by SI-exo can also be inhibited by the AKT/NFκB agonist. Finally, through the *in vivo* experiment of the GO mouse model, we further determined the relationship between exosomes’ mir-885-3p sequence, AKT/NFκB signaling pathway, and glucocorticoid sensitivity. As a conclusion, plasma exosomes deliver mir-885-3p and inhibit the AKT/NFκB signaling pathway to improve the glucocorticoid sensitivity of OFs. Exosome mir-885-3p can be used as a biomarker to determine the sensitivity of ivGCs in GO patients.

## Introduction

Graves ophthalmopathy (GO) is an organ-specific autoimmune disease associated with Graves’ disease (1). 25%–50% of Graves’ disease is accompanied by varying degrees of GO. GO significantly affects the appearance of patients. In severe cases, the patient’s eyeball is fixed, resulting in corneal ulcer, total ophthalmia, and even blindness. Even mild GO will also lead to a significant decline in the psychological state and quality of life (2).

IvGCs is the first-line treatment for moderate-to-severe active GO. For mild GO, ivGCs is also recommended when the quality of life is seriously affected (3). In other words, most of active GO needs ivGCs treatment. However, ivGCs is only effective in 70%–80% of GO patients (3, 4), which means that at least one GO patient is insensitive to ivGCs in five patients. Insensitive patients who choose 12-week ivGCs not only delay treatment but also bear the risk of adverse reactions of glucocorticoids, such as cortical hyperfunction, severe infection, and organ dysfunction. Therefore, it is very important to find an appropriate method to determine the ivGCs sensitivity of GO patients.

Studies found that the clinical signs of GO patients (5), the duration of eye symptoms and restoration of euthyroidism (6), the response to ivGCs during treatment (7), ocular MRI alone (8, 9), or combined with clinical activity score (CAS) (10) may be helpful to determine the sensitivity of ivGCs. However, some of these methods are subjective and cannot be quantified, and some are expensive and difficult to popularize. Moreover, the time of judging sensitivity by these methods is relatively lagging. Therefore, there is still a lack of effective biomarkers that are convenient to detect and determine the sensitivity of ivGCs before making treatment plans.

The biomarkers that can predict the sensitivity of ivGCs in GO patients mainly include proteins, nucleic acids, and lipids. It is generally believed that the nucleotide sequence change of GR can change the sensitivity of glucocorticoid sensitivity. However, this phenomenon seems not to be found in GO patients treated with ivGCs. A study of 58 GO patients treated with ivGCs found that the 3 GR gene polymorphisms, ER22/23EK, N363s, and BCL1 do not influence the therapeutic effect of steroids (11). Li found that patients with high IgG4 levels responded better to ivGCs treatment (12). However, this does not mean that patients with normal or low IgG4 levels are less sensitive to ivGCs. It has also been confirmed that patients with baseline LDL >193.6 mg/dl may have poor response to ivGCs treatment (13). However, this phenomenon can be ameliorated by lipid-lowering therapy. Studies have shown that serum miR-224-5p may predict whether GO patients are sensitive to ivGCs (14).

Exosomes are 30–150-nm membrane vesicles secreted by almost all types of human cells. In 1983, exosomes were first found in sheep reticulocytes. After nearly 40 years of research, exosome detection technologies are becoming more and more mature, and independent databases of exosomes such as exoRBase (http://www.exorbase.org/exoRBaseV2/toIndex) and exocorta (http://exocarta.org/index.html) were established. However, serum miRNAs are fragile and very sensitive to RNA enzymes in the air, and its level will fluctuate with physical factors. Under the protection of the double-layer membrane structure, plasma exosome miRNAs remain stable without degradation. More importantly, exosome miRNAs can be easily and stably extracted even in long-term frozen samples. Plasma exosome miRNAs may be helpful to determine the sensitivity of ivGCs.

Therefore, the purpose of this study is to search for exosome miRNAs that can early determine the sensitivity of ivGCs in GO patients and to clarify the mechanism of exosome miRNAs regulating glucocorticoid sensitivity. In order to solve this problem, miRNA sequencing was performed on plasma exosomes of SI and NSI patients, and differential expression of exosome miRNAs was analyzed. Candidate miRNAs were screened according to the function of target genes and validated by PCR of the extended cohort. We found that plasma exosome mir-885-3p levels were upregulated in ivGCs-sensitive GO patients. Finally, through *in vitro* and *in vivo* functional analyses of exosome mir-885-3p, we confirmed that SI-exo delivered mir-885-3p into OFs and upregulated the level of mir-885-3p. The upregulated mir-885-3p targeted the inhibition AKT/NFκB signaling pathway and improved the glucocorticoid sensitivity of OFs.

## Materials and Methods

### Patients

A total of 17 patients diagnosed as moderate-to-severe activity GO according to NOSPECS score (**Table S1**) and CAS score (**Table S2**) were included. The above patients were from the Department of Endocrinology and Metabolic Diseases of the Second and Fourth Affiliated Hospitals of Harbin Medical University. According to EUGOGO (3), ivGCs was their first choice of treatment. The exclusion criteria included the following: 1) inactive or mildly active GO; 2) patients who received orbital radiation therapy, orbital decompression, or other immunosuppressive therapy within the last 3 months; and 3) contraindications of glucocorticoid therapy.

Baseline plasma was obtained before treatment. After 12 weeks of treatment, according to the grouping criteria of enrolled patients (**Table 1**), the patients were divided into SI (n = 11) and NSI (n = 6) groups. This experiment was reviewed by the Ethics Committee of the Second Affiliated Hospital of Harbin Medical University, China (no. KY2016-046).

**Table 1:** Grouping criteria of enrolled patients.

	Indicator
1	Exophthalmos decreased by at least 2 mm.
2	The width of palpebral fissure is reduced by at least 3 mm.
3	Gorman diplopia score decreased by at least one grade.
4	Any two items in the NOSPECS score reduced by two grades.
5	Visual acuity improved, and visual acuity chart improved by at least one line.

SI: patients whose CAS score is reduced by at least two points and finally less than 3 points, and who have more than two of the above indicators at the same time; NSI: patients with CAS reduced by less than 2 points or still in active stage; patients with no more than 2 improvements in the above indicators.

### Exosome Isolation and Identification

Exosomes were isolated from plasma according to the instructions for the Plasma Exosome Extraction Kit (Invitrogen, Carlsbad, CA, USA). The particle size distribution and concentration of exosomes were detected by a NOT analyzer (Zeta View PMX 110, Particle Metrix, Meerbusch, Germany). Exosome morphology was observed by transmission electron microscopy (Hitachi, Tokyo, Japan). Exosome protein concentrations were determined by BCA protein quantification (Beyotime, Shanghai, China). Finally, exosome-specific marker proteins CD9, CD81, and TSG101 were detected by Western blot.

### Screening Differentially Expressed Exosome miRNAs

Three patients in both SI and NSI groups were randomly selected for exosome miRNA sequencing. Sequencing data were analyzed, and the differentially expressed exosome miRNAs were defined as miRNAs with p value <0.05 and log2(fold change) ≠ 0.

The target genes of miRNA identified in differential exosomes were predicted by miRanda (15)and RNAhybrid (16), and the intersection of target genes was obtained by the two methods for subsequent analysis. The target gene was enriched and analyzed by Kyoto Encyclopedia of Genes and Genomes (KEGG) and Gene Ontology (Go) to predict the function of the target gene. One KEGG pathway and two Go function target genes related to glucocorticoid pharmacological action were selected, and the intersection was taken to obtain the candidate miRNA target genes. Corresponding miRNAs were screened according to target genes. Finally, expanded cohort verification of differential miRNA was performed on the remaining 11 secrete samples.

### Plasmid Construction

The possible sites of mir-885-3p predicting AKT intervention were queried in the miRNA database. Two pairs of DNA single strands (**Table S3**) were designed according to the sequence around the miRNA-binding prediction site gctgcca. After annealing, the DNA single strand was linked to pmirglo (Promega, Madison, WI, USA) plasmid digested with sac I and Xho I, and AKT2 3-UTR and mut-AKT2 3-UTR plasmids were constructed.

### Establishment and Treatment of the GO Animal Model

The GO animal model was established by Balb/c female mice aged 8–10 weeks. The establishment method of the model group was the same as our previous study (17), and the control group was given the same amount of PDF. After modeling, 3 mice in the model group and control group were randomly sacrificed. The left orbital tissue of each mouse was used for histological analysis, and the right orbital tissue was resected under aseptic conditions for primary culture OFs.

Other mice in the model group were randomly divided into 6-week treatment group, 9-week treatment group, 12-week treatment group, and corresponding control group. In the first 6 weeks of treatment, mice in the treatment group were given methylprednisolone 1 mg/kg intraperitoneal injection once a week. From the 7th week, the dose of methylprednisolone was adjusted to 0.5 mg/kg. The control group was given the same amount of normal saline. The mice were sacrificed at the 6th, 9th, and 12th weeks of treatment, and their plasma and orbital tissue were collected for analysis. All mice were provided by the Experimental Animal Center of the Second Affiliated Hospital of Harbin Medical University, raised in an environment free of specific pathogens and operated in accordance with the humane animal care standards stipulated by Harbin Medical University. Animal experiment was reviewed by the Ethics Committee of the Second Affiliated Hospital of Harbin Medical University, China (no. KY2017-110).

### Primary Cell Culture and Identification

GO mouse extraocular muscle was obtained under aseptic conditions, and primary OFs were cultured. The OFs were cultured in Dulbecco’s modified Eagle medium (Corning, Tewksbury, MA, USA) with 10% fetal bovine serum (ExCell Bio, Montevideo, Uruguay) and incubated in a constant 37°C, 5% CO_2_-humidified environment. The cells were identified by immunofluorescence.

### Immunofluorescence Staining of Exosomes and Cells

500 μg purified exosome suspension was taken and diluted to a volume of 500 μl with sterile 1× PBS. According to the instructions, exosomes were labeled with PKH26 staining kit (Maokangbio, Shanghai, China). After staining, the concentration of the stained exosome suspension was quantified by the BCA method (Beyotime, Shanghai, China), and then aseptic 1× PBS adjusted the exosomes to the appropriate concentration.

5 × 10^4^ OFs were spread to a 24-well plate containing sterile slides. Cells were adjusted to a bottling rate of 50%, and a complete culture medium containing 25 μg/ml PKH26-labeled exosomes was added for 24 h. After PBS cleaning, fixation was performed with 4% formaldehyde solution, room temperature peroxidation with 0.5% Triton X-100 solution (Solarbio, Beijing, China), cytoskeleton staining with FITC working solution (Sigma, San Francisco, CA, USA), and nucleus staining with DAPI (Solarbio, China). Finally, they were observed and photographed by a fluorescence microscope.

### miRNA Transfection

The day before transfection, OFs were inoculated into T-25 culture vials in a quantity of 5 × 10^5^ per vial. 10-μl miRNA plasmids (**Table S3**) and Lipofectamine 3000 (Invitrogen, USA) were diluted in the medium and mixed. Each mixed medium was added to OFs for incubation, and the culture was continued for 8 h in the incubator. Complete medium was replaced and expanded to the number required for the experiment.

### Western Blotting

The proteins of suspensions OFs, exosomes, and tissues were obtained and quantified by the BCA method (Beyotime, Shanghai, China). Protein lysates were transferred to the PVDF membrane by 8% SDS-PAGE (Beyotime Biotechnology, China). The PVDF membrane was cleaned with TBST and sealed with 5% skim milk powder for 2 h. The cut film was incubated overnight in the primary antibody dilution solution and 2 h in the secondary antibody dilution solution. Finally, the film was scanned. The main antibodies used included HRP-binding GAPDH monoclonal antibody (ProteinTech, Wuhan, China), AKT polyclonal antibody (ProteinTech, China), NFκB P65 polyclonal antibody (ProteinTech, China), Rabbit Anti-phosphorylated NFκB P65 (Ser276) Antibody (Bioss, Beijing, China), Rabbit Anti-phosphorylated AKT (Ser473) antibody (Bioss, China), IL-1α polyclonal antibody (ProteinTech, China), ICAM1 polyclonal antibody (ProteinTech, China), CD9 monoclonal antibody (ProteinTech, China), TSG101 polyclonal antibody (ProteinTech, China), CD81 monoclonal antibody (ProteinTech, China), Goat Anti-mouse IgG (H+L) (ProteinTech, China), Goat anti-rabbit IgG (H+L) (ProteinTech, China), and glucocorticoid receptor polyclonal antibody (ProteinTech, China).

### Real-Time Quantitative PCR

TRIzol (Thermo Fisher Scientific, Waltham, MA, USA) extracted total RNA from OFs, exosomes, and tissues. 1 µg RNA was reversed transcribed using the transcript first-strand cDNA synthesis Kit (Roche Diagnostics GmbH, Mannheim, Germany). Thermal cycling conditions were as follows: pre-denaturation at 95°C for 3 min, 95°C for 20 s, and 60°C for 45 s for 40 cycles. Fluorescence was measured at the end of each cycle. Primers were synthesized by Shanghai Tianhao Biotechnology Co., Ltd., China. Detailed sequences information of primers are shown in **Table S4**.

### Double Luciferase Activity Detection

The OFs transfected with pGRE-luc (Beyotime, Shanghai, China) and pRL-TK (Beyotime, Shanghai, China) were incubated with lysate, and 300-μl samples were taken into the chemiluminescence detection tube. 500 μl 1× firefly luciferase reaction solution (Solebo Technology Co., Ltd., China) was added to the reaction tube. The Junior LB 9509 chemiluminescence detector detected the luminescence value for 10 s. 500 μl 1× Aquin luciferase reaction solution (Sobibor Technology Co., Ltd., China) was added and gently blown and mixed. The luminescence value was detected by a Junior LB 9509 chemiluminescence detector for 10 s. Relative luciferase activity was calculated based on the two luminescence obtained in each group.

## Results

### Compared With NSI, Plasma Exosome mir-885-3p Was Upregulated, and mir-4474-3p and mir-615-3p Were Downregulated in SI Patients

**Figure 1:**
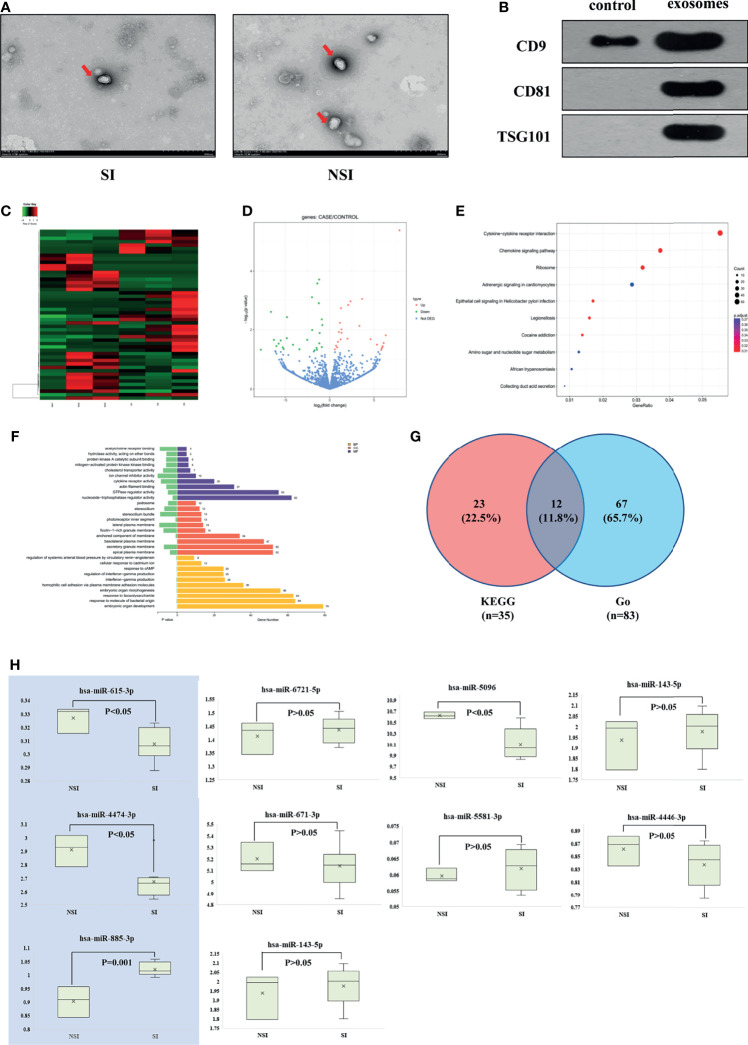
Screening differentially expressed exosome miRNAs. **(A)** Transmission electron microscopy of plasma exosomes of the SI and NSI groups. **(B)** Western blot detected exosome-labeled proteins CD9, CD81, and TSG101. **(C)** Heat map of the cluster analysis of differentially expressed miRNAs among 6 random samples from the SI and NSI groups. **(D)** Volcano map of differentially expressed miRNAs among 6 random samples from the SI and NSI groups. **(E)** Scatter plot of KEGG enrichment in differential exosome miRNA target genes. **(F)** Bar chart of Go enrichment analysis of differential exosome miRNA target genes. **(G)** Veen analysis of differential exosome miRNA target genes. **(H)** PCR results of candidate exosome miRNAs among the remaining samples.

In this study, three patients in the SI and NSI groups were randomly selected to extract plasma exosomes (hereinafter referred to as SI-exo, NSI-exo). Transmission electron microscopy, Western blot, and nanoparticle tracking analysis (NTA) were used to verify that the extract was plasma exosome. Transmission electron microscopy showed that there was no significant difference in the morphology of plasma exosomes between SI and NSI, with a diameter of about 100 nm and a typical concave disc-like structure (**Figure 1A**). Western blot showed a positive expression of plasma exosome-specific marker proteins CD9, CD81, and TSG101 (**Figure 1B**). NTA analysis showed that the vesicles at about 100 nm in the SI and NSI groups accounted for more than 95% (**Figure S1**). These results indicated that exosomes were extracted successfully from plasma exosomes. In addition, the BCA quantification of plasma exosome protein in SI and NSI groups was 1.51 ± 0.02 and 1.52 ± 0.01 mg/ml, respectively. There was no statistical difference between the two groups, indicating that there was no significant difference in the concentration of exosome between the two groups.

Subsequently, miRNA expression profiles of 6 plasma exosomes were analyzed by miRNA-seq. A total of 50 differentially expressed exosome miRNAs were screened. Compared with the NSI group, there were 26 upregulated miRNAs and 24 downregulated miRNAs in the SI group (**Figures 1C, D**). The scatter plot of KEGG enrichment (**Figure 1E**) showed the 10 most significant KEGG enrichment signaling pathways, including the cytokine–cytokine receptor interaction, chemokine signaling pathway, ribosome signaling pathway, amino sugar and nucleotide sugar metabolism, etc. 35 target genes enriched in chemokine signaling pathways related to GC pharmacological action were selected for the follow-up study. Bar chart of Go enrichment analysis is shown in **Figure 1F**. A total of 83 Go target genes enriched in cytokine receptor activity and reaction to lipopolysaccharide were selected. The 83 miRNAs and 35 target genes screened from KEGG were intersected, and 12 differentially expressed exosome miRNAs target genes were obtained: *CCR7/CX3CR1/CXCR2/CXCR3/CXCR5/AKT1/CCL5/CXCL1/CXCL2/CXCL3/MAPK3/NFκB1* (**Figure 1G**).

The above 12 target genes correspond to 10 candidate miRNAs. Compared with the NSI group, there were 6 miRNAs upregulated (*miR-6721-5p/miR-5096/miR-4446-3p/miR-885-3p/miR-4433b-3p/miR-671-3p*) and 4 miRNAs downregulated (*miR-615-3p/miR-4474-3p/miR-143-5p/miR-5581-3p*) in the SI group. The levels of the above miRNA in plasma exosomes of the remaining 11 patients were detected by PCR. Compared with the NSI group, the expression of mir-885-3p was highly expressed (*p* = 0.001), and mir-4474-3p (*p* < 0.05) and mir-615-3p (*p* < 0.05) were low expressed (**Figure 1H**) in exosomes of the SI group. KEGG functional enrichment results showed that *AKT*, the predicted target gene of mir-885-3p, was a classic molecule of the chemokine signaling pathway. NFκB was a downstream protein of the chemokine signaling pathway, and it could regulate the expression of GR to some extent. Meanwhile, the difference of mir-885-3p between the two groups was the most significant (*p* = 0.001). Thus, mir-885-3p was selected for follow-up mechanism research.

### SI-exo Improves the GC Sensitivity of OFs

In the above experiments, we observed that patients with upregulation of plasma exosome mir-885-3p at baseline were sensitive to ivGCs. However, it was still unclear whether there was a clear causal relationship and intrinsic association between them. To explore the exact mechanism, it was necessary to confirm that the increased glucocorticoid sensitivity was caused by exosomes.

**Figure 2:**
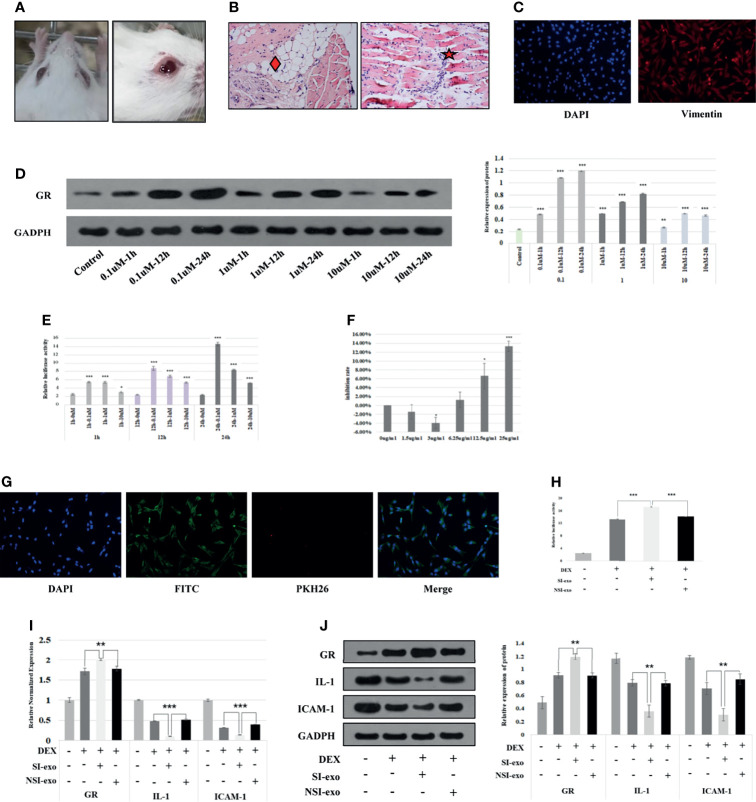
SI-exo improves the glucocorticoid sensitivity of OFs. **(A)** Eye pictures of GO mice. **(B)** HE staining of retrobulbar tissue of GO mice (SP*200); “♦”: fat infiltration; “★”: inflammatory cell infiltration. **(C)** Results of primary OFs immunofluorescence identification; DAPI: labeling the OF nucleus with blue fluorescence; Vimentin: labeling vimentin with red fluorescence. **(D)** The Western blot result of the GR protein expression level when OFs in DEX at 0.1, 1, and 10 μM for 1, 12, and 24 h, respectively. Compared with the control group: *p < 0.05; **p < 0.01; ***p < 0.001. **(E)** The GRE luciferase reporter plasmid activity result when OFs in DEX at 0.1, 1, and 10 μM for 1, 12, and 24 h. Compared with the 0-μM group: *p < 0.05; **p < 0.01; ***p < 0.001. **(F)** CCK8 results of OFs in exosomes at 1, 1.5, 3, 6.25, 12.5, and 25 μg/ml for 24 h. Compared with the 0-μg/ml group: *p < 0.05; ***p < 0.001. **(G)** Immunofluorescence results of coculture of plasma exosomes and OFs. DAPI: labeling the OF nucleus with blue fluorescence; PKH26: labeling plasma exosomes with red fluorescence; FITC: labeling cytoskeleton with green fluorescence. **(H)** Plasmid activity of GRE luciferase reporter gene results in each group. DEX: 0.1 μm DEX-treated OFs for 24 h; SI-exo/NSI-exo: 25 μg/ml SI-exo/NSI-exo-treated OFs for 24 h; *p < 0.05; **p < 0.01; ***p < 0.001. **(I)** Statistical chart of PCR detection of GR, IL-1, and ICAM-1 mRNA levels in each group. Grouping and statistical methods are the same as **(I)**. **(J)** Western blot detection of GR, IL-1, and ICAM-1 protein levels and statistical chart in each group. Grouping and statistical methods are the same as **(I)**.

At first, we cultured primary OFs from GO mice and identified them and then screened the optimal DEX, exosome intervention concentration, and time of OFs. The GO mice used in this project were derived from our recently reported study (17). It could be seen that the eyes of the mice in the model group are obviously prominent, bulbar conjunctival edema, tears, and increased periocular secretions (**Figure 2A**). Pathological results showed the fat infiltration between the optic nerve and muscles and the extraocular myositis cell infiltration of the model mice (**Figure 2B**). The primary culture of OFs was performed on GO mouse extraocular muscle. The immunofluorescence results showed that vimentin staining was positive, confirming that the extracted cells were OFs (**Figure 2C**). We treated with OFs at 0.1, 1, and 10 μM DEX conditions for 1, 12, and 24 h, respectively. It could be seen that the OFs under 24-h treatment with 0.1 μM DEX and the GR expression level were the highest (**Figure 2D**), and GRE luciferase reporter plasmid activity was the highest (**Figure 2E**). Therefore, the intervention concentration and time point of DEX were selected as 0.1 μM for 24 h. Subsequently, we used exosomes with protein concentrations of 0, 1.5, 3, 6.25, 12.5, and 25 μg/ml to intervene OFs 24 h. CCK8 found that exosomes had the greatest influence on cells under the condition of 25 μg/ml acting on OFs for 24 h. Therefore, 25 μg/ml for 24 h was selected as the intervention concentration and time point of exosomes (**Figure 2F**).

In order to explore the effect of exosome miRNAs on OFs, it was necessary to confirm that exosomes could enter into OFs. We cocultured 25 μg/ml plasma exosomes labeled with PKH26 (red fluorescence under the microscope) with OFs for 24 h. The merged image of immunofluorescence showed that the plasma exosomes were endocytosed by OFs (**Figure 2G**). Subsequently, we explored the effect of exosomes on the glucocorticoid sensitivity of OFs. We treated OFs with 25 μg/ml SI-exo and NSI-exo at 0.1 μM DEX for 24 h. It was found that SI-exo significantly upregulated the GRE luciferase reporter plasmid activity of OFs compared with NSI-exo (*p* < 0.001) (**Figure 2H**). At the same time, after SI-exo treatment, GR protein and mRNA levels were significantly upregulated (*p* < 0.01), and the protein (*p* < 0.01) and mRNA (*p* < 0.001) levels of inflammatory factors interleukin-1 (IL-1) and intercellular cell adhesion molecule-1 (ICAM-1) were significantly downregulated (**Figures 2I, J**). It could be seen that SI-exo can improve the glucocorticoid sensitivity of OFs.

### SI-exo Improves the Glucocorticoid Sensitivity of OFs by Transferring mir-885-3p

**Figure 3:**
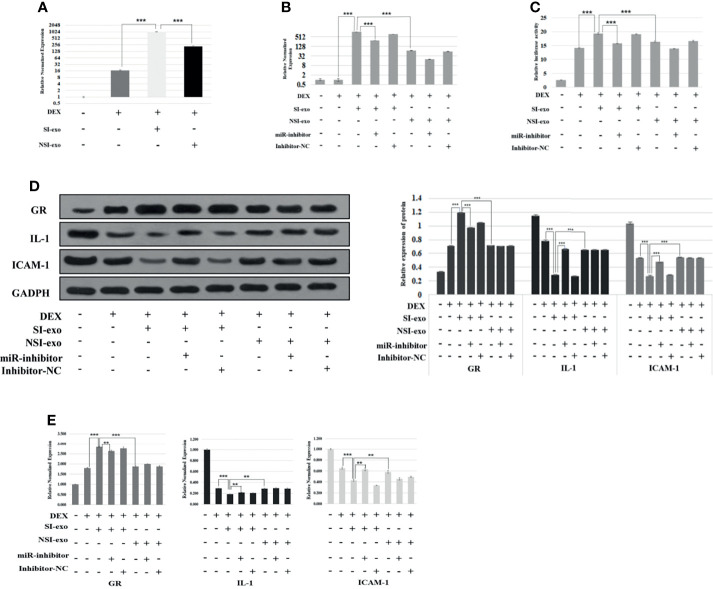
SI-exo improves the glucocorticoid sensitivity of OFs by transmitting mir-885-3p. **(A, B)** Statistical chart of PCR detection of mir-885-3p in each group. DEX: 0.1 μm DEX-treated OFs for 24 h; SI-exo/NSI-exo: 25 μg/ml SI-exo/NSI-exo-treated OFs for 24 h; miR-885-3p: miR-885-mic-treated OFs for 24 h; miR-nc: NC miRNA-treated OFs for 24 h; **p < 0.01; ***p < 0.001. **(C)** Plasmid activity of GRE luciferase reporter gene results in each group; grouping and statistical methods are the same as in **(B)**; **(D)** Western blot detection of GR, IL-1, and ICAM-1 protein levels in each group; grouping and statistical methods are the same as in **(B)**. **(E)** Statistical chart of PCR detection of GR, IL-1, and ICAM-1 mRNA levels in each group; grouping and statistical methods are the same as in **(B)**.

Previous experiments had confirmed that SI-exo could improve the glucocorticoid sensitivity of OFs, but it still cannot prove that the regulation of SI-exo on glucocorticoid sensitivity was achieved by changing the level of miR**-**885-3p in OFs. In the following experiments, we confirmed the above hypothesis. We treated OFs with 25 μg/ml SI-exo and NSI-exo, respectively, under 0.1 μM DEX for 24 h. We found that both SI-exo and NSI-exo could upregulate the level of mir-885-3p of OFs. Compared with NSI-exo, SI-exo had a stronger upregulation effect on the level of mir-885-3p of OFs (*p* < 0.001) (**Figure 3A**). Subsequently, we added the miR-inhibitor and inhibitor NC respectively on the basis of the above grouping. It was observed that when the SI-exo and miR-inhibitors intervened in cells at the same time, the level of mir-885-3p was significantly lower than that of SI-exo alone under the condition of 0.1 μM DEX (*p* < 0.001). The same trend results also appeared in the NSI-exo group. This indicates that the miR inhibitor can inhibit well the upregulation of miR-885-3p by exosomes (**Figure 3B**). We also observed the GRE luciferase reporter gene plasmid activity and GR, IL-1, ICAM-1 protein, and mRNA levels in each group. It was found that under the condition of 0.1 μM DEX, compared with that treated with SI-exo alone, the GRE luciferase reporter gene plasmid activity of OFs treated with SI-exo and miR inhibitor was significantly decreased (*p* < 0.001) (**Figure 3C**), the GR protein and mRNA levels were significantly downregulated (*p* < 0.001), and inflammatory factor IL-1 and ICAM-1 protein levels (*p* < 0.001) and mRNA levels (*p* < 0.01) were significantly upregulated (**Figures 3D, E**). In other words, when the upregulation of SI-exo on miR-885-3p was blocked by the miR inhibitor, the effect of SI-exo on improving OF glucocorticoid sensitivity was weakened. That is, SI-exo enhances the glucocorticoid sensitivity of OFs by transferring miR-885-3p.

### Mir-885-3p Upregulates Glucocorticoid Sensitivity of OFs

**Figure 4:**
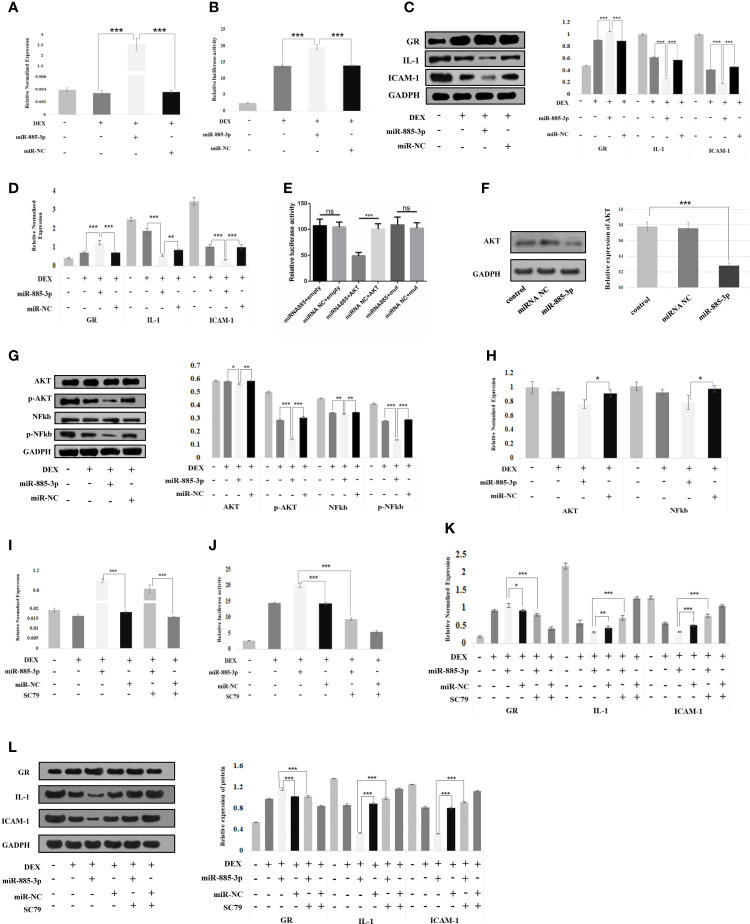
Mir-885-3p regulated the glucocorticoid sensitivity of OFs by targeting AKT/NFκB. **(A)** Statistical chart of PCR detection of mir-885-3p in each group. DEX: 0.1 μm DEX-treated OFs for 24 h; miR-885-3p: miR-885-mic-treated OFs for 24 h; miR-NC: NC miRNA-treated OFs for 24 h; *p < 0.05; **p < 0.01; ***p < 0.001. **(B)** Plasmid activity of GRE luciferase reporter gene results in each group; grouping and statistical methods are the same as in **(A)**. **(C)** Western blot detection of GR, IL-1, and ICAM-1 protein levels in each group; grouping and statistical methods are the same as in **(A)**. **(D)** Statistical chart of PCR detection of GR, IL-1, and ICAM-1 mRNA levels in each group; grouping and statistical methods are the same as in **(A)**. **(E)** The results of double luciferase in 293T cells; miRNA-885: miRNA-885-3p mimics co-transfected with plasmid; miRNA NC: corresponding NC miRNA co-transfected with plasmid; empty: co-transfected plasmid was empty; AKT: co-transfected plasmid was AKT2 3′-UTR recombinant plasmid; mut: co-transfected plasmid was mut-AKT2 3′-UTR recombinant plasmid; ns, no statistical significance; ***p < 0.001. **(F)** Western blot detection of AKT protein levels in each group; grouping and statistical methods are the same as in **(A)**. **(G)** Western blot detection of AKT, pAKT, NFκB, and pNFκB protein levels in each group; grouping and statistical methods are the same as in **(A)**. **(H)** Statistical chart of PCR detection of AKT and NFκB mRNA levels in each group; grouping and statistical methods are the same as in **(A)**. **(I)** Statistical chart of PCR detection of mir-885-3p in each group. DEX: 0.1 μm DEX-treated OFs for 24 h; miR-885-3p: miR-885-mic-treated OFs for 24 h; miR-NC: NC miRNA-treated OFs for 24 h; SC79: AKT agonist-treated OFs for 24 h; *p < 0.05; **p < 0.01; ***p < 0.001. **(J)** Plasmid activity of GRE luciferase reporter gene results in each group; grouping and statistical methods are the same as in **(I)**. **(K)** Statistical chart of PCR detection of GR, IL-1, and ICAM-1 mRNA levels in each group; grouping and statistical methods are the same as in **(I)**. **(L)** Western blot detection of GR, IL-1, and ICAM-1 protein levels in each group; grouping and statistical methods are the same as in **(I)**.

We attempted to explore the relationship between miR-885-3p and glucocorticoid sensitivity. For this purpose, mir-885-mimics and mir-NC were used to treat OFs under 0.1 μM DEX for 24 h. It was found that mir-885-3p levels were upregulated after the addition of mir-885-3p mimics, which confirmed the good upregulation effect of mir-885-mimics on mir-885-3p (**Figure 4A**). Then, we detected the GRE luciferase reporter gene plasmid activity and GR, IL-1, ICAM-1 protein, and mRNA levels of each group of OFs. It was found that when mir-885-3p was upregulated, GRE gene plasmid activity increased (*p* < 0.001) (**Figure 4B**), the GR level was significantly upregulated (*p*<0.001), and inflammatory factors were significantly downregulated (**Figures 4C, D**). The above results suggested that mir-885-3p upregulates the glucocorticoid sensitivity of OFs.

### Mir-885-3p Upregulates Glucocorticoid Sensitivity of OFs by Regulating AKT/NFκB

In the following experiments, we explored the endogenous association between mir-885-3p upregulation and increased the glucocorticoid sensitivity. In the previous target gene prediction of differentially expressed exosome miRNAs, we predicted that mir-885-3p might be able to target the regulation of *AKT*. At the same time, *AKT* was also one of the 12 target genes of differential exosome miRNA. Therefore, we constructed AKT2 3′-UTR and mut-AKT2 3′-UTR plasmids by using *AKT* as the downstream target gene of mir-885-3p. 293T cells and OFs were co-transfected with miRNA mimics, miR-NC, recombinant plasmid, and empty plasmid. Compared with the control group, the luciferase activity of the 293T cells co-transfected with mir-885-mimics and AKT2 3-UTR recombinant plasmid was significantly decreased after transfection 48 h (*p* < 0.001) (**Figure 4E**). Western blot results showed that the AKT level of OFs significantly decreased after transfection of mir-885-mimics (*p* < 0.001) (**Figure 4F**). The targeted regulation of *AKT* by mir-885-3p was confirmed.

Subsequently, we transfected mir-885-mimic and mir-NC under 0.1-μM DEX conditions and treated OFs for 24 h. Western results showed that the AKT, pAKT, NFκB, and pNFκB protein levels of OFs were changed, especially pAKT (*p* < 0.001) and pNFκB (*p* < 0.001) levels which were significantly downregulated (**Figure 4G**) after transfection with mir-885-mimic compared with that of mir-NC. The trend of AKT and NFκB mRNA levels in each group was consistent with Western blot results (**Figure 4H**). It was confirmed that upregulated mir-885-3p regulates the AKT/NFκB signaling pathway.

On the basis of the above grouping, we added AKT agonist SC79 as mir-885-SC79 group and mir-NC-SC79 group, respectively. We found that the mir-885-3p level in the mir-885-SC79 group was significantly higher than that in the mir-NC-SC79 group after the addition of SC79, indicating that SC79 did not affect the upregulation of mir-885-3p by the mir-885-mimic (**Figure 4I**). Analysis of GRE luciferase reporter gene plasmid activity showed that the GRE gene plasmid activity in the mir-885-3p group was significantly higher than that in the DEX group and the mir-NC group (*p* < 0.001), but the GRE gene plasmid activity was significantly decreased after the addition of SC79 (*p* < 0.001) (**Figure 4J**). Similarly, the protein and mRNA levels of GR in the mir-885 group were significantly higher than those in the mir-NC group (*p* < 0.001), and the protein and mRNA levels of inflammatory factors IL-1 and ICAM-1 were lower than those in the Mir-NC group (*p* < 0.001). After the addition of SC79, the protein and mRNA levels of GR, IL-1, and ICAM1 showed an opposite trend compared with the mir-885-3p group (**Figures 4K, L**). These results suggested that mir-885-3p regulates glucocorticoid sensitivity through the AKT/NFκB pathway.

### SI-exo Transmits mir-885-3p to Target AKT/NFκB Upregulates the Glucocorticoid Sensitivity of OFs

**Figure 5:**
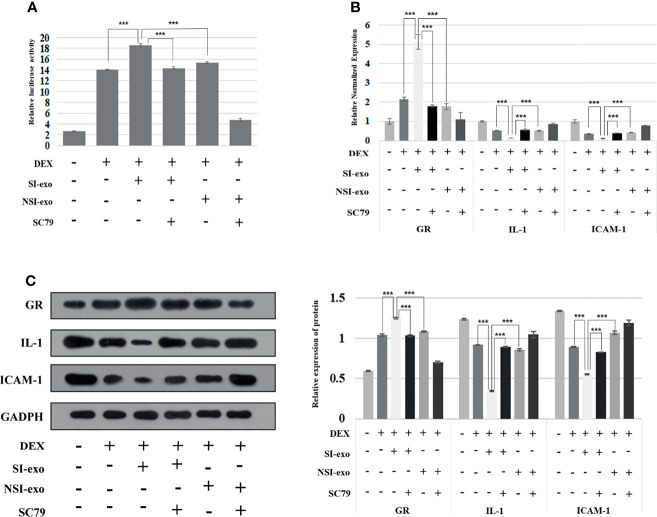
SI-exo transfer miR-885-3p targeting AKT/NFκB improving the glucocorticoid sensitivity of OFs. **(A)** Plasmid activity of GRE luciferase reporter gene results in each group. DEX: 0.1 μm DEX-treated OFs for 24 h; SI-exo/NSI-exo: 25 μg/ml SI-exo/NSI-exo-treated OFs for 24 h; SC79: AKT agonist-treated OFs for 24 h; ***p < 0.001. **(B)** Statistical chart of PCR detection of GR, IL-1, and ICAM-1 mRNA levels in each group; grouping and statistical methods are the same as in **(A)**. **(C)** Western blot detection of GR, IL-1, and ICAM-1 protein levels in each group; grouping and statistical methods are the same as in **(A)**.

**Figure 6:**
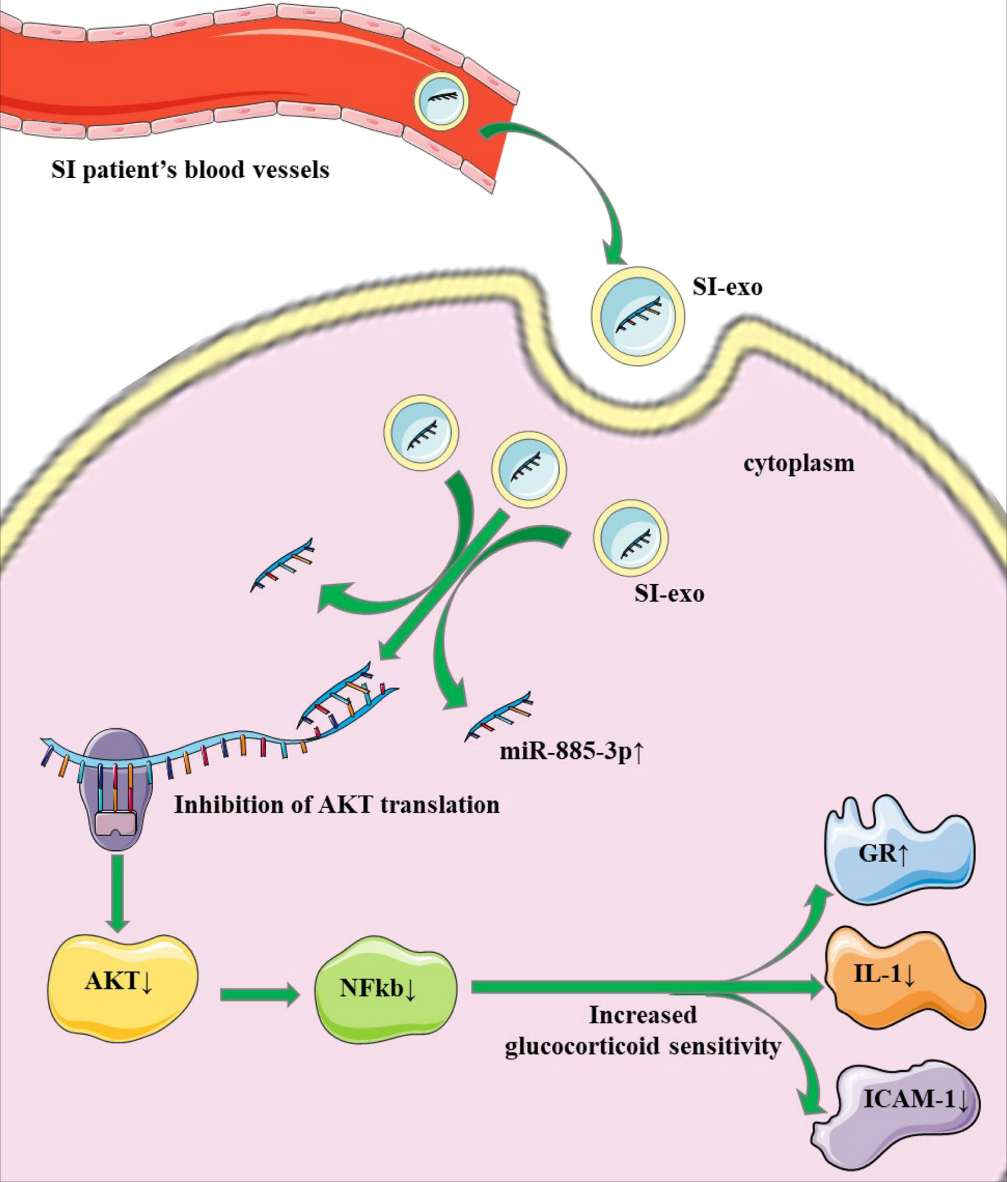
Mechanism pattern of exosome mir-885-3p improving the glucocorticoid sensitivity of OFs.

In the above experiment, we confirmed that mir-885-3p delivery by SI-exo increases the glucocorticoid sensitivity of OFs and upregulated mir-885-3p targeting regulates AKT/NFκB and improves the glucocorticoid sensitivity of OFs. Next, we attempted to link the two conclusions together, confirming that mir-885-3p delivered by SI-exo also improved the glucocorticoid sensitivity of OFs by regulating AKT/NFκB. Under 0.1-μM DEX conditions, SC79 was added into OFs treated with SI-exo and NSI-exo. Compared with OFs treated with SI-exo alone, the plasmid activity of the GRE luciferase reporter gene in the OFs treated with both SI-exo and SC79 decreased significantly (*p* < 0.001) (**Figure 5A**), the levels of GR protein and mRNA decreased significantly (p < 0.001), and the levels of protein and mRNA of inflammatory factors IL-1 and ICAM-1 increased significantly (*p* < 0.001). The same trend results also appeared in the NSI-exo group (**Figures 5B, C**). In other words, when the AKT/NFκB signaling pathway was activated, the effect of exosomes on OF glucocorticoid sensitivity weakened. In conclusion, SI-exo delivered mir-885-3p into OFs, upregulated the OF mir-885-3p level, targeted the inhibition AKT/NFκB signaling pathway, and improved the glucocorticoid sensitivity of OFs (**Figure 6**).

### The Relationship Between Exosome mir-885-3p Sequence, AKT/NFκB, and Glucocorticoid Sensitivity Exists *In Vivo*

In previous studies, we elucidated the intrinsic association between plasma exosomes with high mir-885-3p levels and glucocorticoid sensitivity. However, the results of *in vitro* experiments could not fully represent the mechanism *in vivo*. Therefore, GO mice were treated with glucocorticoid and sacrificed at the 6th, 9th, and 12th weeks of the treatment course, respectively, to observe whether the relationship between mir-885-3p sequence and AKT/NFκB and glucocorticoid sensitivity existed *in vivo*.

**Figure 7:**
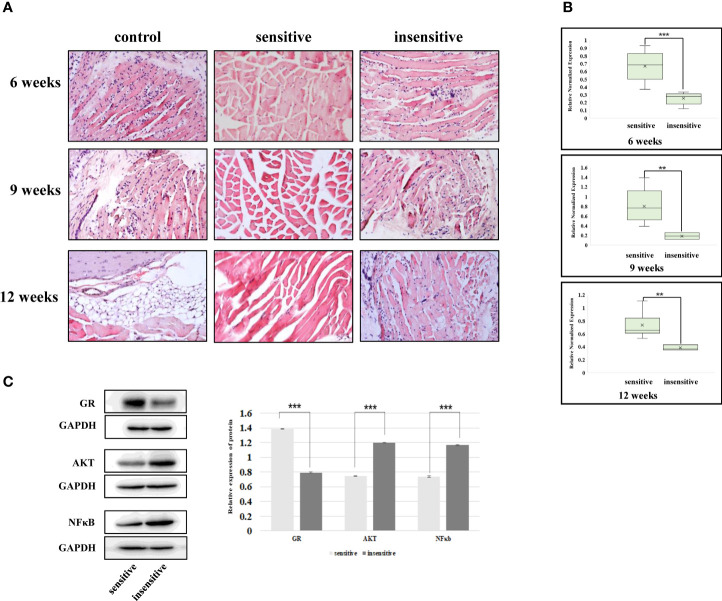
The relationship between exosomes’ mir-885-3p sequence, AKT/NFκB, and glucocorticoid sensitivity exists *in vivo*. **(A)** HE staining results of GO mice in the 6th, 9th, and 12th weeks of treatment. **(B)** The level of mir-885-3p sequence in plasma exosomes of mice in the 6th, 9th, and 12th weeks of treatment; **p < 0.01; ***p < 0.001. **(C)** Western blot detection of GR, AKT, and NFκB protein levels; ***p < 0.001.

The mice sacrificed at each time point were divided into glucocorticoid-sensitive group and insensitive group by orbital tissue histopathology. At the 6th week of treatment, nearly half of the mice had poor response, and at weeks 9 and 12, the proportion of mice with poor response decreased (**Table 2**). HE staining results of the orbital tissues in different groups of mice at each time point showed infiltration of inflammatory cells and connective tissue in the control group and the insensitive group. In contrast, muscle fibers in the sensitive group were smooth, and no such changes were observed in the interfascicles (**Figure 7A**). The sequence levels of plasma exosome mir-883-3p in each group of mice in **Table 2** were detected, and it was found that compared with the glucocorticoid-insensitive group, the sequence levels of plasma exosome mir-883-3p in the glucocorticoid-sensitive group were upregulated at week 6 (*p* < 0.01), week 9 (*p* < 0.01), and week 12 (*p* < 0.001) of the treatment course (**Figure 7B**). Finally, we detected the protein levels of GR, AKT, and NFκB in the orbital tissues of the two groups at 12 weeks after treatment. Compared with the insensitive mice, GR expression in the orbital tissues of the sensitive group was upregulated (*p* < 0.001), while AKT (*p* < 0.001) and NFκB (*p* < 0.001) were downregulated (**Figure 7C**). These results suggested that the relationship between the level of mir-885-3p sequence in exosome, AKT/NFκB, and glucocorticoid sensitivity still existed *in vivo*.

**Table 2:** Statistical table of mouse grouping.

Group	6 week(n)	9 week(n)	12 week(n)
Control	2	2	3
Sensitive	6	3	3
Insensitive	7	11	9
Total	15	16	15

## Discussion

As the first-line treatment for GO, ivGCs has a sensitivity rate of only 70%–80%. At present, there is still a lack of biomarkers to effectively predict the curative effect of ivGCs. In this study, biomarkers that can predict the sensitivity of ivGCs before the formulation of diagnosis and treatment were explored by clinical sample sequencing, cell functional experiments, and *in vivo* validation of animal models, and the mechanism of their regulation of ivGCs sensitivity was elucidated.

Exosomes maintain normal physiological processes by acting on target cells and play an important role in the occurrence and development of diseases (18, 19). It has long been established that plasma exosomes are closely related to pathophysiological processes in the body. However, since Salomon et al. found in 2016 that the plasma exosome concentration changes with gestation age and status (20), the discussion on whether the relationship between exosomes and body is caused by the change in the concentration of exosome itself or the change of its contents has been continuing (20–22). In this study, we quantified the protein concentration of exosomes in SI and NSI patients, and the results showed that there was no significant difference in plasma exosomes concentration between the two groups. This suggested that the differences in miRNA levels detected in subsequent sequencing and validation results were caused by changes in corresponding miRNA content in exosomes, rather than changes in exosome concentration per unit volume of plasma.

MiRNAs are known to be associated with many diseases (23–25). In this study, miRNA sequencing technology was used to comprehensively analyze the miRNA expression profiles of ivGCs sensitive and insensitive patients. 50 differentially expressed exosome miRNAs were obtained by bioinformatics. In order to further explore the functions of these exosome miRNAs and screen target miRNAs related to glucocorticoids, we performed KEGG and Go enrichment analyses of different miRNA target genes. The chemokine signaling pathway enriched by KEGG is closely related to the pharmacological action of glucocorticoids (26, 27). The downstream protein of the chemokine signaling pathway is NFκB, and the anti-inflammatory effect of glucocorticoids is mainly achieved through the transcriptional inhibition of NFκB by GR (14, 28). At the same time, NFκB can regulate the expression of GR to some extent (29). Studies have shown that chemokines can affect the central nervous system response to reactive glucocorticoids by mediating T cell directional migration (30), and GR signal transduction caused by endogenous glucocorticoid rhythm changes can also lead to rhythmic fluctuations in chemokine receptor levels (31). Based on the above evidence, 35 differential exosome miRNA target genes enriched by KEGG into the chemokine signaling pathway were selected for follow-up analysis in this study. Go enrichment analysis results showed that differential exosome miRNA target genes were significantly enriched in cytokine receptor activity and response to lipopolysaccharide, which were closely related to the mechanism of glucocorticoids (32–36). Studies have found that increased stress of lipopolysaccharide can lead to GR exon variation (35), and the increase in ITS content is positively correlated with the expression of GR-β (36). GR itself acts as a cytokine receptor, and its ability to respond to glucocorticoids is closely related to cytokine receptor activity. Therefore, 83 target genes enriched in the above two Go functions were selected in this study, and the intersection with the above 35 target genes was obtained to obtain 12 differentially expressed exosome miRNA target genes, which correspond to 10 candidate miRNAs. According to Go and KEGG functional enrichment results, miRNA target genes differentially expressed in SI and NSI groups were mostly enriched in glucocorticoid-related pathways. Thus, the differential exosome miRNAs screened in this study are closely related to ivGCs sensitivity.

To further clarify miRNAs associated with ivGCs sensitivity, 10 candidate exosome miRNAs were verified by qRT-PCR in the extended cohort. Mir-885-3p, mir-4474-3p, and mir-615-3p showed significant differences between the two groups, which can be used as potential biomarkers to determine the sensitivity of ivGCs in GO patients. Based on statistical differences and miRNA functions, mir-885-3p was selected for follow-up study. To sum up, we only observed that patients with upregulation of plasma exosome mir-885-3p at baseline were sensitive to ivGCs. However, whether there is a clear causal relationship between them and the exact mechanism are still unclear. To explore the relationship between them, we performed cytological function analysis of SI-exo.

Studies have shown that specific miRNAs delivered by exosomes can affect the function of cocultured cells (19, 37). These results indicate that miRNAs in plasma exosomes can be transported to recipient cells through exosomes and then regulate functions of cells, although the parental cells of plasma exosomes selected in this study are not clear. However, exosome miRNA transmission is not a random process; exosomes play a role by binding with target cells through specific mechanisms (38). Therefore, at first, we confirmed that exosomes can be successfully ingested by OFs and upregulated mir-885-3p levels of OFs. These results suggest that plasma exosomes deliver mir-885-3p into OFs.

Next, we investigated the effect of plasma exosomes which transported mir-885-3p on the glucocorticoid sensitivity of OFs. For this purpose, four indicators were selected in this study to reflect the glucocorticoid sensitivity of cells. The IvGCs treatment of Graves ophthalmopathy is mainly because of its anti-inflammatory effects. The mechanism of its receptor GR is in the form of the inactive OF cytoplasm. After the glucocorticoid enters the cells, the cells within the passive diffusion combined with GR form the receptor and the ligand complexes to nuclear transfer, combined with the target gene promoter sequences of GRE, inhibiting inflammatory cytokine gene transcription (39). Therefore, the sensitivity of cells to glucocorticoid can be reflected by the transcriptional activation level of GRE, GR expression level, and inflammatory factor level. In this study, we found that when SI-exo was ingested by OFs, the GRE luciferase reporter gene plasmid activity, GR protein, and mRNA levels were significantly increased, and the levels of inflammatory factors decreased correspondingly. In other words, the glucocorticoid sensitivity of OFs increased. However, when mir-885-3p delivered by SI-exo was inhibited by the mir inhibitor, the effect of SI-exo on the glucocorticoid sensitivity of OFs was weakened. These results confirm that SI-exo enhances the glucocorticoid sensitivity of OFs by delivering mir-885-3p.

Subsequently, we attempted to find the internal relationship between mir-885-3p and OF glucocorticoid sensitivity. Prior to this, we first need to confirm that changes in mir-885-3p levels affect OF glucocorticoid sensitivity. In this study, it was found that when mir-885-3p was upregulated in OFs, the plasmid activity of GRE luciferase reporter gene and the GR protein and mRNA levels were significantly increased. The levels of IL-1 and ICAM-1 protein and mRNA decreased correspondingly. These results confirmed that the high level of mir-885-3p can increase the glucocorticoid sensitivity of OFs.

Therefore, what is the pathway through which mir-885-3p regulates glucocorticoid sensitivity? Exosome miRNAs can downregulate target protein levels by regulating posttranscriptional translation (40). Current studies have found that mir-885-3p can regulate the functions of multiple target genes such as *HOXB2* (41), *TLR4* (42), and *Aurora A* (43). Among the 12 differential exosome miRNAs target genes obtained by KEGG and Go (*CCR7/CX3CR1/CXCR2/CXCR3/CXCR5/AKT1/CCL5/CXCL1/CXCL2/CXCL3/MAPK3/NFκB1*), we predicted that mir-885-3p targeted the regulation of *AKT*. As a classical molecule of the chemokine signaling pathway, AKT regulates the expression of downstream protein NFκB (44), which is consistent with KEGG functional enrichment results. Therefore, we hypothesized that the mir-885-3p-targeted regulation of the AKT/NFκB signaling pathway enhances the glucocorticoid sensitivity of OFs.

The luciferase activity of mir-885-3p mimics and the AKT2 3′-UTR recombinant plasmid group was significantly reduced by dual luciferase reporter gene assay, confirming that mir-885-3p can target the 3′-UTR of *AKT*. In addition, to further verify the regulatory relationship, Western blot analysis showed that the AKT protein level of OFs was significantly downregulated after transfection with mir-885-3p. These results jointly confirmed that mir-885-3p can target and inhibit AKT expression. Subsequently, we found that when the AKT/NFκB pathway was excited by SC79, the promoting effect of mir-885-3p on glucocorticoid sensitivity disappeared. In other words, mir-885-3p enhances the glucocorticoid sensitivity of OFs by target regulation of AKT/NFκB.

For the sake of rigor, we further investigated whether mir-885-3p delivered by SI-exo also plays a role through AKT/NFκB. The results showed that when the AKT/NFκB pathway was activated by SC79, the promoting effect of mir-885-3p delivered by SI-exo on glucocorticoid sensitivity was also weakened. That is, mir-885-3p delivered by SI-exo improved the glucocorticoid sensitivity of OFs *via* AKT/NFκB.

It is worth mentioning that we found that the NSI-exo group also showed the same trend as SI-exo. NSI-exo can also upregulate the level of mir-885-3p in OFs and affect the levels of GR, IL-1, and ICAM-1. This effect can also be inhibited by the miR-inhibitor. This is because NSI-exo also contains a small amount of miR-885-3p. This also confirmed the effect of exosome mir-885-3p on the glucocorticoid sensitivity of OFs to some extent. However, since miR-885-3p in NSI-exo is not as high as in SI-exo, the effect of NSI-exo on OFs is limited. Similar phenomena have been seen in clinical cases. After ivGCs treatment, NSI group patients also showed a slight improvement. In other words, NSI group patients also have little response to ivGCs. We speculate that these responses are related to the small amount of mir-885-3p in plasma exosomes of NSI group patients. However, these slight improvements do not mean that ivGCs treatment completely alleviates the condition of NSI patients. It also does not mean that NSI patients are sensitive to ivGCs. This may be related to the insufficient amount of mir-885-3p in plasma exosomes of patients with NSI. It can also confirm our conclusion to some extent.

However, the mechanism described above was found *in vitro*. It remains unclear whether the mechanism holds true *in vivo*. In the patients we collected in this study, neither the SI group nor the NSI group received surgical treatment. Therefore, we have no way to obtain the patients’ posterior tissue to verify the above mechanism. We solve this problem through animal experiments. We treated GO mice with glucocorticoid and observed the relationship between mir-885-3p sequence level in plasma exosomes, AKT/NFκB level in orbital tissues, and glucocorticoid sensitivity in mice, confirming that our conclusions still hold *in vivo*.

We treated GO mice with glucocorticoid, and the mice were sacrificed at the 6th, 9th, and 12th weeks of the course, respectively. The mice were divided into glucocorticoid-sensitive group and insensitive group according to the pathological manifestations of orbital tissues histopathology. At the 6th week of treatment, about half (6/13) of mice were insensitive to glucocorticoid, while at the 9th and 12th weeks, the proportion of insensitive mice decreased to 3/14 and 3/12, respectively. The proportion of glucocorticoid-insensitive mice was similar at weeks 9 and 12. This result is consistent with the report by Guia that the sensitivity of ivGCs can be judged according to the response of GO patients during 6–8 weeks of treatment (5), which also confirms the accuracy of the results of this study. This suggests that glucocorticoid sensitivity in GO mice may be determined by performance at week 9 of treatment.

Due to the large amount of plasma required for extraction of exosomes and the small body weight of mice, it cannot ensure that mice can still survive when enough plasma is collected. Therefore, the existing technique in this study was unable to detect baseline plasma exosome mir-883-3p sequence levels in mice. We supported the conclusion of this study by detecting the level of plasma exosome mir-883-3p sequence in mice at the 6th, 9th, and 12th weeks of treatment. It was found that compared with the insensitive group, plasma exosome mir-885-3p sequence and GR level were upregulated, and AKT and NFκB levels were downregulated in the sensitive group after glucocorticoid treatment, which was consistent with the results *in vitro* experiment. We confirm that the changes of exosome mir-885-3p sequence, AKT/NFκB, and glucocorticoid sensitivity still existed *in vivo*.

## Conclusion

In conclusion, this study provided the effective plasma exosome miRNA expression profile of GO patients by miRNA sequencing and searched stable and easily detected circulating biomarkers of ivGCs sensitivity. Meanwhile, the mechanism of exosome mir-885-3p regulating the sensitivity of ivGCs was elucidated: SI-Exo transfer mir-885-3p targeting the AKT/NFκB signaling pathway improves the ivGCs sensitivity of GO patients. Plasma exosome mir-885-3p is expected to become a reliable and feasible biomarker to predict GO patients’ sensitivity of ivGCs. It provides a scientific basis for the selection of treatment methods for GO patients and is of great significance to ensure the good prognosis of patients.

## Data Availability Statement

The original contributions presented in the study are publicly available. These data can be found here: https://www.ncbi.nlm.nih.gov/geo/query/acc.cgi?acc=GSE190515.

## Ethics Statement

The studies involving human participants were reviewed and approved by the Ethics Committee of the Second Affiliated Hospital of Harbin Medical University, China (no. KY2016-046). The patients/participants provided their written informed consent to participate in this study. The animal study was reviewed and approved by the Ethics Committee of the Second Affiliated Hospital of Harbin Medical University, China (no. KY2017-110).

## Author Contributions

HQ conceived the idea of this manuscript. JS performed the experiment. JW, YZ, JjL, JL, JY, MG, and JH collected the data and performed the data analysis. JS wrote the manuscript. All authors contributed to the article and approved the submitted version.

## Funding

This work was supported by the National Natural Science Fund of China (grant nos. 81872560 and 82073491).

## Conflict of Interest

The authors declare that the research was conducted in the absence of any commercial or financial relationships that could be construed as a potential conflict of interest.

## Publisher’s Note

All claims expressed in this article are solely those of the authors and do not necessarily represent those of their affiliated organizations, or those of the publisher, the editors and the reviewers. Any product that may be evaluated in this article, or claim that may be made by its manufacturer, is not guaranteed or endorsed by the publisher.

## Supplementary Material

Supplementary Figure 1 NTA analysis of plasma exosomes. Percentage: the proportion of vesicles with different sizes.
