# Supplementary figures and images for: Plasma Exosomes Transfer miR-885-3p Targeting the AKT/NFκB Signaling Pathway to Improve the Sensitivity of Intravenous Glucocorticoid Therapy Against Graves Ophthalmopathy
Source: Front Immunol. 2022 Feb 21;13:819680. doi: 10.3389/fimmu.2022.819680 (PMC8900193; doi:10.3389/fimmu.2022.819680)

## SI-exo

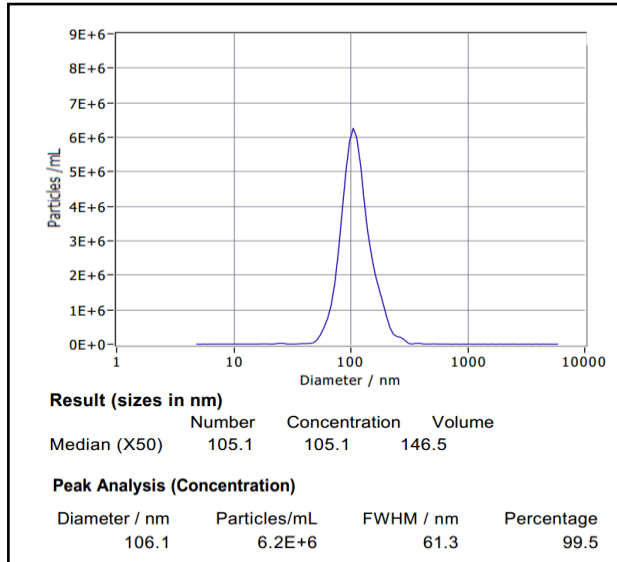

## NSI-exo

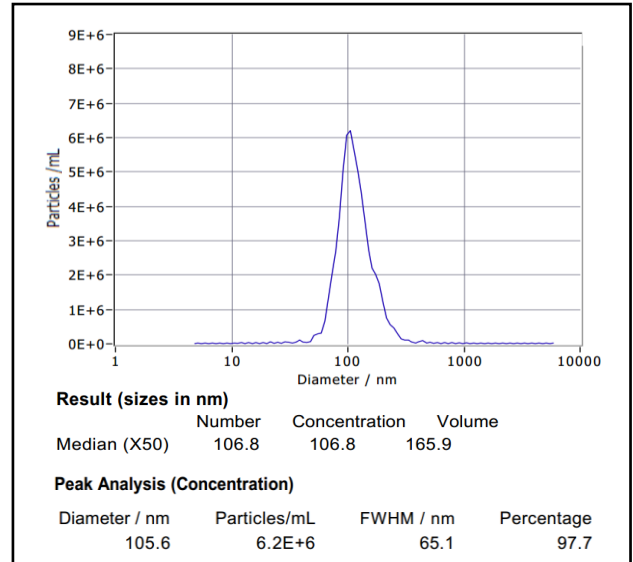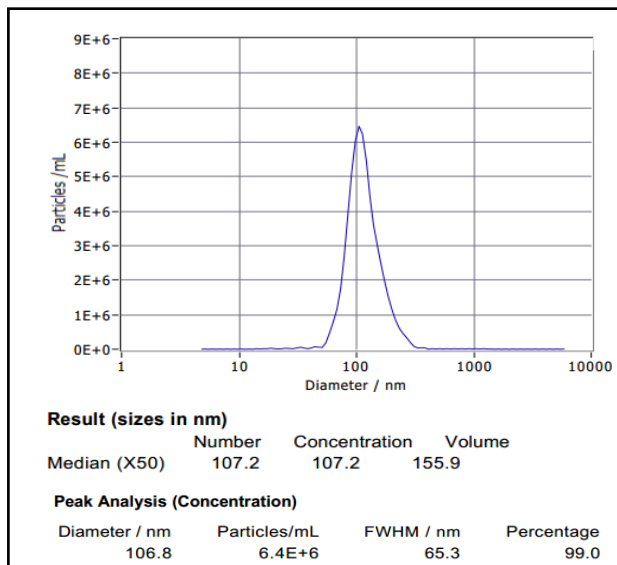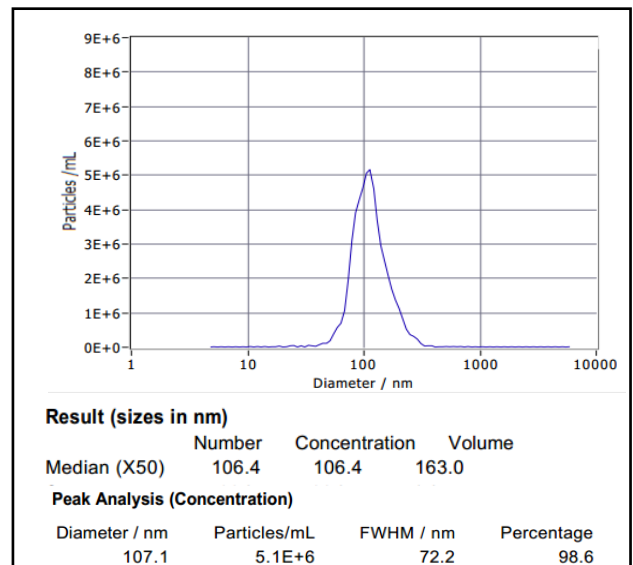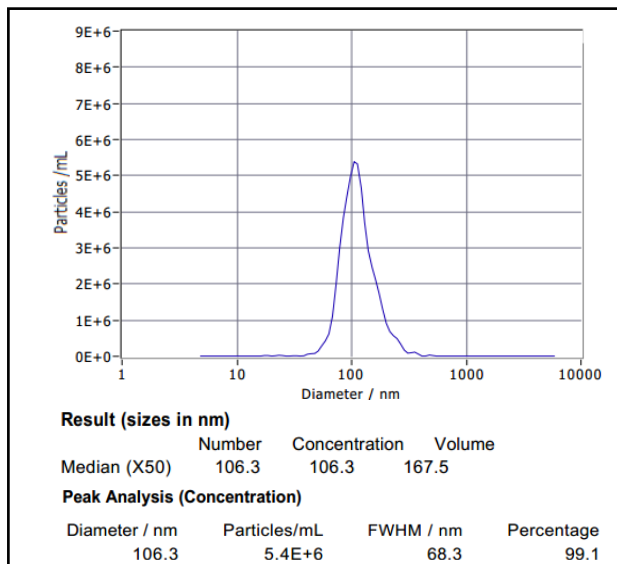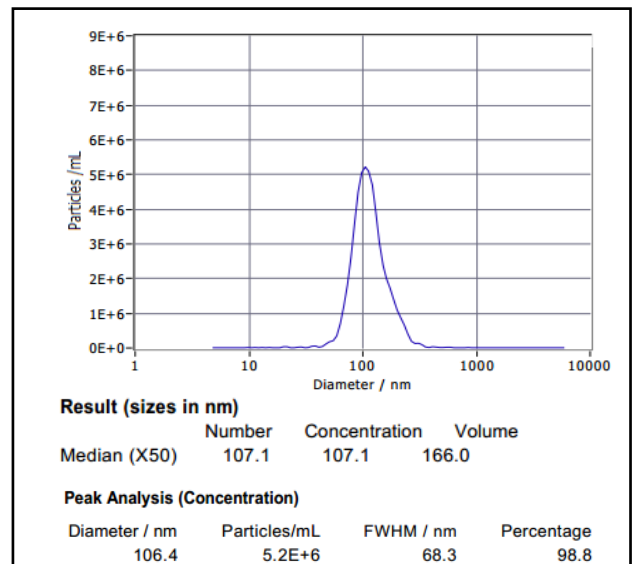

Supplement: Supplementary Figure 1 — NTA analysis of plasma exosomes. Percentage: the proportion of vesicles with different sizes. [file DataSheet_1.pdf]
