# Supplementary material for: Plasma Exosomes Transfer miR-885-3p Targeting the AKT/NFκB Signaling Pathway to Improve the Sensitivity of Intravenous Glucocorticoid Therapy Against Graves Ophthalmopathy
Source: Front Immunol. 2022 Feb 21;13:819680. doi: 10.3389/fimmu.2022.819680 (PMC8900193; doi:10.3389/fimmu.2022.819680)
Supplement: Supplementary file 2 [file Table_1.docx]

**Table S1. GO NOSPECS Scoring Criteria**

| **Classification** | **Abbreviation** | **Definition** |
| --- | --- | --- |
| 0 | N | no signs or symptoms |
| 1 | O | only signs |
| 2 | S | soft-tissue involvement |
| 3 | P | proptosis |
| 4 | E | extraocular muscle involvement |
| 5 | C | corneal involvement |
| 6 | S | sight loss |

Table note: GO can be diagnosed if it is required to reach grade 2 or above; Not all GO courses develop from grade 0 to grade 6.
