# Supplementary material for: Plasma Exosomes Transfer miR-885-3p Targeting the AKT/NFκB Signaling Pathway to Improve the Sensitivity of Intravenous Glucocorticoid Therapy Against Graves Ophthalmopathy
Source: Front Immunol. 2022 Feb 21;13:819680. doi: 10.3389/fimmu.2022.819680 (PMC8900193; doi:10.3389/fimmu.2022.819680)
Supplement: Supplementary file 3 [file Table_2.docx]

**Table S2. Graves ophthalmopathy clinical activity score assessment**

| **Symptom** | **Score** |
| --- | --- |
| Spontaneous retrobulbar pain | 1 |
| Pain on attempted upward or downward gaze | 1 |
| Redness of eyelids | 1 |
| Redness of conjunctiva | 1 |
| Swelling of caruncle or plica | 1 |
| Swelling of eyelids | 1 |
| Swelling of conjunctiva (chemosis) | 1 |

Table note: Active GO:CAS≥3 points; Inactive GO:CAS＜3 points.
