# Supplementary material for: Plasma Exosomes Transfer miR-885-3p Targeting the AKT/NFκB Signaling Pathway to Improve the Sensitivity of Intravenous Glucocorticoid Therapy Against Graves Ophthalmopathy
Source: Front Immunol. 2022 Feb 21;13:819680. doi: 10.3389/fimmu.2022.819680 (PMC8900193; doi:10.3389/fimmu.2022.819680)
Supplement: Supplementary file 4 [file Table_3.docx]

**Table S3. Sequence information.**

| **Item** | **Sequence** |
| --- | --- |
| AKT2 3'-UTR | CGCTAGGTGACAGCGTGTTAATgctgccaCCATGAATGAGGTATCTGTCATCAAAGAAGGCTGGCTCCC |
|  | TCGAGGGAGCCAGCCTTCTTTGATGACAGATACCTCATTCATGGtggcagcATTAACACGCTGTCACCTAGCGAGCT |
| mut-AKT2 3'-UTR | CGCTAGGTGACAGCGTGTTAATctgatcgCCATGAATGAGGTATCTGTCATCAAAGAAGGCTGGCTCCC |
|  | TCGAGGGAGCCAGCCTTCTTTGATGACAGATACCTCATTCATGGcgatcagATTAACACGCTGTCACCTAGCGAGCT |
| miR-885-3p mimics | Sense AGGCAGCGGGGUGUAGUGGAUA |
|  | Antisense UAUCCACUACACCCCGCUGCCU |
| miR-NC mimics | Sense UUGUACUACACAAAAGUACUG |
|  | Antisense GUACUUUUGUGUAGUACAAUU |
| miR-885-3p inhibitor | Sense UAUCCACUACACCCCGCUGCCU |
| miR-inhibitor-NC | Sense CAGUACUUGUGUGUAGUACAAC |
