# Supplementary material for: Plasma Exosomes Transfer miR-885-3p Targeting the AKT/NFκB Signaling Pathway to Improve the Sensitivity of Intravenous Glucocorticoid Therapy Against Graves Ophthalmopathy
Source: Front Immunol. 2022 Feb 21;13:819680. doi: 10.3389/fimmu.2022.819680 (PMC8900193; doi:10.3389/fimmu.2022.819680)
Supplement: Supplementary file 5 [file Table_4.docx]

**Table S4. Primer sequences**

| primer | sequence |
| --- | --- |
| hsa-miR-6721-5p-RT | GTCGTATCCAGTGCAGGGTCCGAGGTATTCGCACTGGATACGACCTCCTA |
| hsa-miR-5096-RT | GTCGTATCCAGTGCAGGGTCCGAGGTATTCGCACTGGATACGACGCCTGA |
| hsa-miR-4446-3p-RT | GTCGTATCCAGTGCAGGGTCCGAGGTATTCGCACTGGATACGACACCCAT |
| hsa-miR-885-3p-RT | GTCGTATCCAGTGCAGGGTCCGAGGTATTCGCACTGGATACGACTATCCA |
| hsa-miR-4433b-3p-RT | GTCGTATCCAGTGCAGGGTCCGAGGTATTCGCACTGGATACGACACGTCC |
| hsa-miR-671-3p-RT | GTCGTATCCAGTGCAGGGTCCGAGGTATTCGCACTGGATACGACGGTGGA |
| hsa-miR-615-3p-RT | GTCGTATCCAGTGCAGGGTCCGAGGTATTCGCACTGGATACGACAAGAGG |
| hsa-miR-4474-3p-RT | GTCGTATCCAGTGCAGGGTCCGAGGTATTCGCACTGGATACGACTTAGCC |
| hsa-miR-143-5p-RT | GTCGTATCCAGTGCAGGGTCCGAGGTATTCGCACTGGATACGACACCAGA |
| hsa-miR-5581-3p-RT | GTCGTATCCAGTGCAGGGTCCGAGGTATTCGCACTGGATACGACGGAACT |
| hsa-miR-24-3p-RT | GTCGTATCCAGTGCAGGGTCCGAGGTATTCGCACTGGATACGACCTGTTC |
| hsa-miR-6721-5p-F | TGGGCAGGGGCTTATTGTAGGAG |
| hsa-miR-5096-F | GTTTCACCATGTTGGTCAGGC |
| hsa-miR-4446-3p-F | CAGGGCTGGCAGTGACATGGGT |
| hsa-miR-885-3p-F | AGGCAGCGGGGTGTAGTGGATAG |
| hsa-miR-4433b-3p-F | CAGGAGTGGGGGGTGGGACGT |
| hsa-miR-671-3p-F | TCCGGTTCTCAGGGCTCCACC |
| hsa-miR-615-3p-F | TCCGAGCCTGGGTCTCCCTCTT |
| hsa-miR-4474-3p-F | TTGTGGCTGGTCATGAGGCTAA |
| hsa-miR-143-5p-F | GGTGCAGTGCTGCATCTCTGGTG |
| hsa-miR-5581-3p-F | TTCCATGCCTCCTAGAAGTTCC |
| hsa-miR-24-3p-F | TGGCTCAGTTCAGCAGGAACAG |
| Stem-loop-uni-R | CCAGTGCAGGGTCCGAGGTATTC |
| GAPDH-F | AGGTCGGTGTGAACGGATT |
| GAPDH-R | TGAGTGGAGTCATACTGGAACA |
| GR-F | CCTGGTGTGCTCCGATGAA |
| GR-R | GCTGTCCTTCCACTGCTCTT |
| IL-1-F | CTGAAGAAGAGACGGCTGAGT |
| IL-1-R | CTGGTAGGTGTAAGGTGCTGAT |
| ICAM-1-F | CACTTCCTCTGGCTGTCACA |
| ICAM-1-R | CCATCCACCGAGTCCTCTTAG |
| AKT-F | CACAGCACCTCAGTCACCTT |
| AKT-R | CCTCACACTAAGCAGCGTAGA |
| NFΚb-F | TACACAGGACCAGGAACAGTT |
| NFΚb-R | TCAGCCTCATAGTAGCCATCC |
| U6-F | CTCGCTTCGGCAGCACATATACT |
| U6-R | ACGCTTCACGAATTTGCGTGTC |
